# FairFlow: A transparency-first framework for verifiable and reproducible bioinformatics

**DOI:** 10.1016/j.array.2026.101150

**Published:** 2026-09

**Authors:** Agata D'Onofrio, Eliseo Martelli, Maurizio Alessandri, Sebastian Bucatariu, Sandro Gepiro Contaldo, Maria Luisa Ratto, Jianli Tao, Beatrice Nuvolari, Isabella Castellano, Andrea Loiacono, Sara Bianchi, Maddalena Arigoni, Alessandro Bertero, Elisa Balmas, Roberto Chiarle, Luca Alessandri

**Affiliations:** a University of Turin, Department of Molecular Biotechnology and Health Sciences, Turin, Italy; b University of Turin, Department of Computer Science, Turin, Italy; c Department of Pathology, Boston Children's Hospital and Harvard Medical School, Boston, MA, USA; d Reproducible Bioinformatics, Via nizza 52, Turin, Italy

**Keywords:** Computational reproducibility, Bioinformatics workflows, Containerization, Docker, Workflow managers, Version control, FAIR data

## Abstract

Bioinformatics analyses depend on external software that itself relies on further programs, or dependencies, each with its own release cycle. Because tools are updated to fix bugs and add features, identical code can produce different results depending on which versions are installed, and incomplete documentation of dependencies and versions therefore undermines reproducibility. Containerization packages software with all its dependencies, but demands technical expertise and lacks a standard operating procedure. Existing workflow managers and container tools have improved reproducibility, yet each works differently and requires specialized knowledge, forcing developers to rebuild equivalent solutions from scratch. Here we present FairFlow, a framework that makes rigorous, reproducible analysis accessible without specialized technical knowledge. Developers describe a pipeline once in a declarative, INI-style specification file written in Baryon, and FairFlow automatically generates ready-to-run interfaces for R, Python, Bash, Galaxy, Nextflow and StreamFlow, each executing the analysis inside a version-locked container. The generated interfaces are self-contained: end users need only Docker and their preferred language. As a proof of concept we reanalyzed a published study in two ways: following the original protocol, which does not account for dependency installation, and with a FairFlow-based script. The first yielded only ∼21% concordance with the original findings, traced to an unspecified sequence-alignment tool version, whereas the FairFlow-based reanalysis achieved 98.8–100% concordance across operating systems and reduced setup from months of troubleshooting to a single Docker installation.

## Highlights

• One.bala file yields R, Python, Bash, Galaxy, Nextflow and StreamFlow REIs.
• Unpinned BLASTN version cut HTGTS junction concordance to 21%.
• FairFlow restored 98.8-100% concordance across operating systems and architectures.
• Generated REIs are self-contained: end users need only Docker and the core program.
• CAR-NGS validates the framework on eight NGS pipelines available on GitHub.

## 1 Introduction

The Reproducibility Project in Cancer Biology, an eight-year effort to replicate key oncology studies [1], revealed that only 2% of papers shared open data and none fully described protocols, highlighting the presence of what was called “reproducibility crisis”. Over the years it was clear that reproducibility was not only an issue in the life science field but it was a general issue applicable to many science domains like Bioinformatics, an interdisciplinary field that develops and applies computational methods to analyze, integrate, and interpret biological data; here reproducibility is especially challenging due to the large number of software tools that must work together within a single analytical pipeline, where even a version change in one of them can lead to substantially different results.

The development of containers and package managers (e.g., Docker [2], Conda [3]), generally improved reproducibility in computer science, but cannot guarantee time-stable environments since incomplete version pinning and dependencies often introduce subtle alterations.

Most bioinformatics tools rely on external libraries (dependencies) automatically installed by a package manager, and even when the main tool is pinned to a specific release, its dependencies may continue to evolve, producing slightly different environments each time the installation is repeated.

Beyond technical challenges, computational reproducibility has direct implications for scientific transparency and integrity [4], as the figures and results published in bioinformatics papers often derive from complex analyses that become impossible to verify (or to scrutinize for potential data manipulation), without access to the underlying data, code, and environment specifications. Computational reproducibility is therefore not merely a technical requirement, but a cornerstone of credible and transparent research.

Several workflow managers, such as Nextflow [5] and Snakemake [6], have improved reproducibility by integrating container technologies like Docker. However, they are designed for complex, multi-step pipelines and require considerable expertise to install and configure, which is not always justified: many analyses do not need a full workflow manager. For these cases, and to lower the entry barrier, R-based wrappers such as rCASC [7] and docker4Seq [8] offer a complementary approach that runs an entire analysis with a single command.

These wrappers, known as front-end functions, compress complex steps within a single command drastically lowering the entry barrier for end-users, allowing researchers to execute an entire analysis by calling a single R or Python function without installing dependencies or configuring environments. Combined with Docker, which captures the entire software stack enabling analyses to be executed with minimal setup, they represent a robust solution to the reproducibility problem [9]. However, creating these front-end wrappers requires substantial manual work at multiple steps of development: developers must first write the core analysis scripts, then build and maintain Docker images with pinned dependencies, and finally implement language-specific wrapper functions that handle parameter validation, file mounting, error management, and cleanup. This lack of flexibility becomes particularly problematic as pipelines grow in complexity: when different laboratories need even slight variations in parameters or execution logic, they cannot simply modify the existing wrapper but must instead create their own customized version from scratch. Over time, this has led to a proliferation of redundant workflows, each solving similar problems but maintained independently and incompatible with one another, fragmenting the bioinformatics ecosystem rather than building upon shared foundations.

While manually built execution interfaces and workflow managers have each advanced reproducibility in different ways, such interfaces combined with containerized environments currently represent one of the most effective solutions (Table 1) for reproducible and accessible bioinformatics, as demonstrated by tools like rCASC and docker4Seq, or by Nextflow process definitions. Yet their adoption remains limited: creating and maintaining these interfaces requires substantial manual effort, each is tightly coupled to a specific programming language, and no unified standard exists that would allow the same pipeline to be deployed across different languages and platforms without rewriting the interface logic from scratch. Here we present FairFlow, a framework that automates exactly this process, supporting both developers and end users in the creation and execution of reproducible pipelines. At its core, FairFlow takes a single declarative specification file, written in Baryon, and automatically generates what we define as **Reproducible Execution Interfaces (REIs)**: user-facing execution artifacts that expose parameters, validate inputs, manage deterministic data mounting, and invoke containerized computational units through a standardized and reproducible layer. From a single Baryon file, FairFlow can generate any of these interface forms: an R function, a Python or Bash script, a Galaxy wrapper, or orchestration-ready configurations for Nextflow or StreamFlow, so the same unit can be run programmatically, from a shell, through a graphical interface, or inside a workflow manager without rewriting any interface logic. Importantly, what a Baryon file describes is a single containerized computational unit, and the developer chooses its scope: in simple cases that unit is an entire pipeline exposed as one command, whereas in complex, multi-step analyses each task is described by its own Baryon file and exposed through its own REIs. FairFlow is therefore not a workflow manager and does not replace these systems: it produces the reproducible, containerized units that such managers orchestrate, and that end users and reviewers can equally run on their own with nothing more than Docker.

**Table 1:** Trade-offs of existing approaches to running a bioinformatics analysis. Comparison of the standard manual approach and containerized single-command wrappers (e.g., rCASC, docker4Seq) across environment setup, execution, flexibility, reproducibility, and ease of extension.

	Standard Approach	Containerized wrapper (e.g. rCASC, docker4Seq)
Environment setup	Every tool and dependency installed by hand	Single container, nothing installed manually
Execution	Each step run manually, in sequence	One command, executed inside one fixed image
Flexibility	Full; every step can be customized	Limited; hard to alter individual steps
Reproducibility	Poor; environment drifts over time and across hardware	High, but tied to a single monolithic image
Ease of extension	Low; manual reconfiguration for each change	Low; the wrapper must be rebuilt to be adapted

We demonstrate FairFlow's effectiveness by reanalyzing a published HTGTS study, where concordance improved from ∼21% to 98.8–100%, and showcase its capabilities through CAR-NGS, an R package containing ready-to-use NGS pipelines.

## 2 Materials and methods

All software, container images, datasets, and test systems used in this study are listed in Supplementary Table S3.

FairFlow is distributed at https://github.com/Fairflow-BioinformaticsFramework.

### 2.1 Limitations of standard containerization approaches

A Docker image is a packaged computational environment that contains an operating system layer, libraries, and executables. Images are usually defined through a Dockerfile, a plain-text recipe listing installation instructions (e.g., FROM, RUN, COPY, ENV) executed sequentially to reproduce the environment. Once built, images are typically pushed to public repositories such as Docker Hub or BioContainers to be reused by others. While this model has revolutionized portability, it still falls short of providing strong reproducibility for scientific workflows.

Pre-built images are transient. Repositories such as Docker Hub may delete or overwrite older versions, and “latest” tags drift over time. Even if an image digest is archived, the original build context may become inaccessible, breaking long-term traceability.

Dockerfiles are under-specified. They often rely on remote resources (apt-get install, pip install, conda install) without fixed versions, checksums, or mirrors. Even when explicit version pinning is applied to top-level packages, their transitive dependencies remain unresolved and may change silently, leading to different binary environments from the same Dockerfile over time.

External dependencies evolve. Package managers inside the build (apt, pip, conda) continuously update their registries, so dependency resolution depends on the moment of build rather than the Dockerfile content.

Hidden layers and base images. A Dockerfile inherits its base from another image (FROM ubuntu:22.04, FROM rocker/rstudio), which itself may change or disappear, causing subtle differences in libraries and compilers even when the visible instructions are identical.

Limited auditability. Dockerfiles do not automatically record full dependency trees, source URLs, or environment digests (SBOM). As a result, two builds with the same recipe may differ in untracked transitive dependencies.

Runtime assumptions. Containers may still depend on host factors such as CPU architecture, kernel version, locale, or filesystem behavior, producing small numerical or formatting differences between systems.

Similarly, Conda environments improve reproducibility but face analogous issues. Conda's dependency solver dynamically resolves versions at installation time using online channel metadata. Because these metadata evolve, running the same environment.yml at different times may produce non-identical environments. Even with version pinning, secondary dependencies can shift, as Conda resolves them opportunistically to satisfy newer constraints. Without explicit lockfiles or offline caches, Conda environments cannot be guaranteed to remain identical across builds.

In summary, standard container and package management approaches improve reproducibility but cannot ensure it. Achieving long-term, auditable reproducibility requires deterministic, fully pinned builds that eliminate online resolution, fix all dependency versions (including the base image), and archive both the environment definition and its binary form in verifiable, content-addressable artifacts.

### 2.2 Ensuring deterministic environments with CREDO

To overcome the inherent instability of standard Dockerfiles and Conda environments, FairFlow employs CREDO [10] (Customizable, REproducible, DOckerfile generator) as its reproducible build backend. CREDO replaces network-dependent builds with a deterministic, two-stage process that guarantees identical environments across systems and time. In the first stage, CREDO installs all declared dependencies in a temporary builder container while recording every version, source, and transitive dependency. All installation artifacts (packages, source files, and metadata) are archived locally in a GitHub-compatible structure. In the second stage, the same instructions are replayed offline, reconstructing the container without accessing external registries or mirrors. This eliminates hidden variability due to package updates, solver drift, or unavailable repositories. The resulting environment is self-contained, auditable, and immutable: every dependency is pinned, every binary has a defined provenance, and the full build context can be redistributed as a TAR archive or referenced by its cryptographic digest. By ensuring that even transitive dependencies are frozen and recoverable, CREDO provides the stable foundation on which FairFlow pipelines are built. Combined with FairFlow's declarative Baryon interface, this architecture turns container reproducibility from a best practice into a guaranteed property—allowing analyses to be rebuilt, verified, and shared with identical results across operating systems, hardware architectures, and time.

### 2.3 FairFlow workflow implementation

**Fig. 1:**
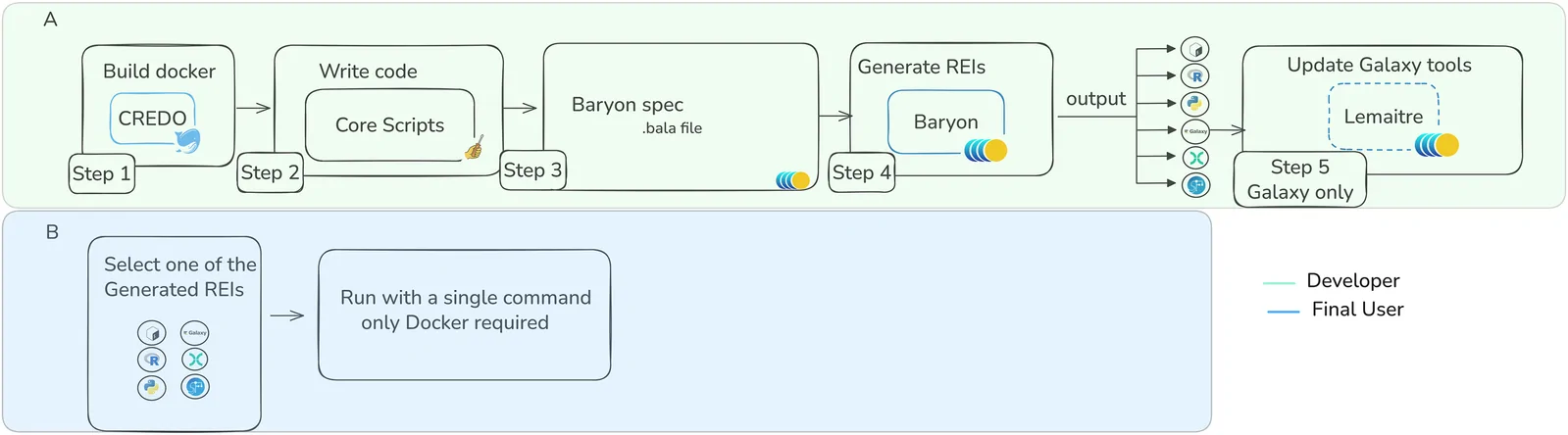
**The FairFlow workflow, from pipeline construction to execution.** (A) Developer side. A pipeline is built in four core steps, plus an optional fifth step for Galaxy deployment: an immutable container image is generated with CREDO (Step 1), the core analysis code is written following the FairFlow coding standard (Step 2), the interface is declared in a Baryon (.bala) specification (Step 3), and the Baryon compiler generates the Reproducible Execution Interfaces (REIs) for the chosen targets (Step 4): R, Python, Bash, Galaxy, Nextflow, and StreamFlow. An optional fifth step deploys the Galaxy tool through Lemaitre (Step 5, Galaxy only). (B) End-user side. The user selects one of the generated REIs and runs the analysis with a single command, requiring only Docker and no installation of dependencies.

The FairFlow framework consists of four main implementation steps (Fig. 1A; see also the online tutorial at https://github.com/Fairflow-BioinformaticsFramework/Tutorial_Fairflow):

Step 1 Container image generation. Immutable Docker containers are built using CREDO to ensure deterministic, time-stable environments. All dependencies (including transitive dependencies) are version-pinned and archived locally. Container images are tagged with cryptographic digests (SHA256) for verifiable reproducibility. On the other hand, another accepted approach is to generate the tar.gz of the file and upload it to Zenodo.
Step 2 Core analysis function development. Analysis functions are written in R, Python, or Bash following FairFlow coding standards: all parameters must be explicitly declared (no implicit defaults), file paths must be handled predictably across systems, functions must not require user interaction during execution, and all functions must return clear error codes.
Step 3 Baryon specification. Pipeline interfaces are defined through.bala files (Baryon specification format, https://github.com/Fairflow-BioinformaticsFramework/baryonlang), which declare: parameters, input files and directories, the container image, and the command-line structure. Baryon specifications are INI-style configuration files that serve as a single source of truth for pipeline interfaces.

A minimal.bala file for the HTGTS pipeline illustrates the declarative syntax:

**Figure:**
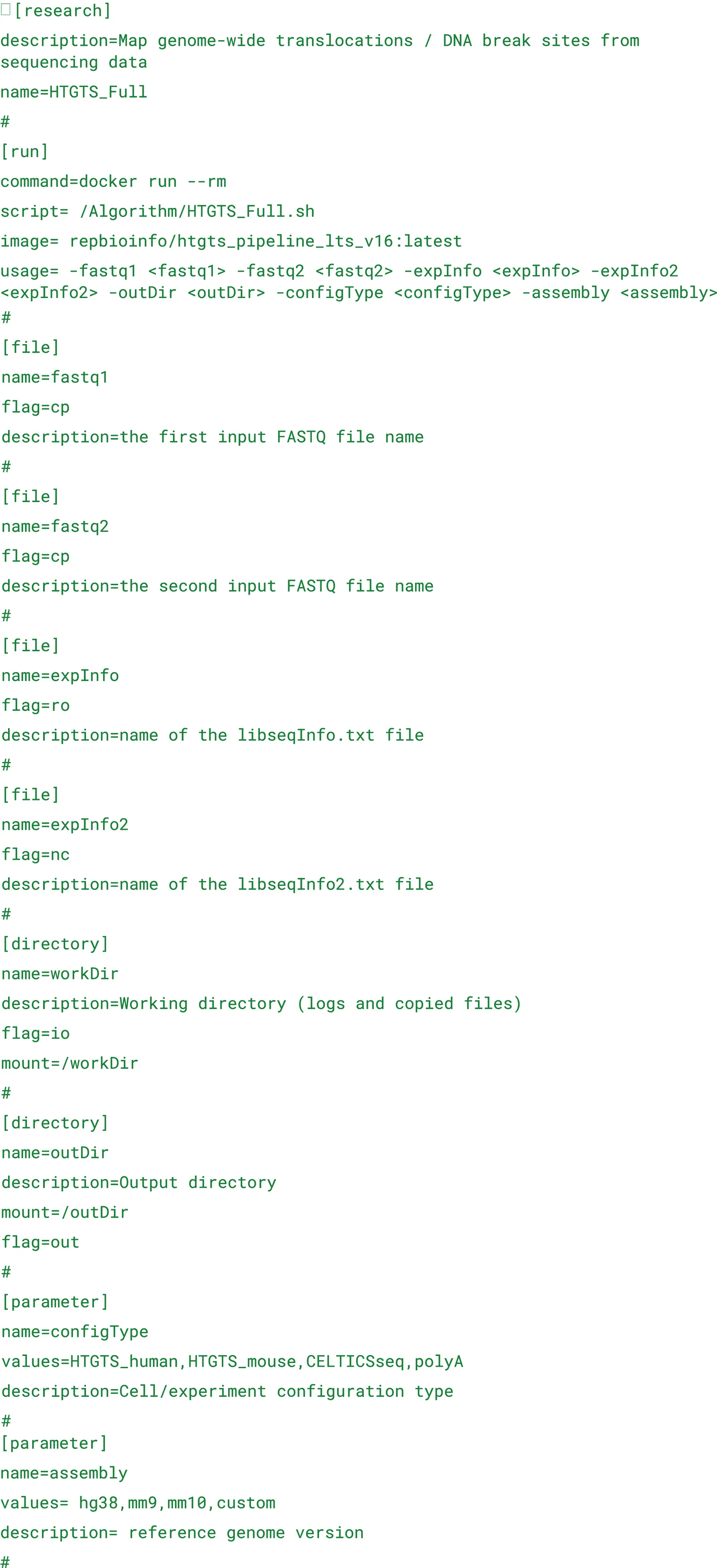

This declarative specification is validated by the Baryon compiler and automatically generates equivalent R, Python, and Bash wrappers that execute the identical containerized pipeline with consistent parameter validation and error handling.

Step 4 REI generation. From each.bala file, the Baryon compiler generates REIs for the requested targets: R wrapper functions for integration into R packages and Python wrapper scripts for command-line or programmatic use (programmatic front-end REIs), Bash scripts for shell-based execution (command-line REIs), Galaxy tool XML definitions for graphical deployment [11] (graphical front-end REIs), and Nextflow or StreamFlow [12] configuration files for use within workflow managers (workflow-ready REIs). A single.bala describes one containerized computational unit, whose scope ranges from an entire pipeline exposed as one command to an individual task within a larger multi-step workflow, and the developer selects which targets to generate. All generated REIs are self-contained and do not require Baryon to be installed at runtime; end users need only Docker and the target language (R, Python, or Bash).

Critically, this modular architecture is designed for extensibility: while FairFlow currently supports these targets, the framework can be expanded by the community to accommodate new programming languages, emerging workflow systems, or specialized execution environments.

### 2.4 Automated wrapper generation enables single-command execution across multiple programming languages and platforms

Once a pipeline is packaged with FairFlow, end users can execute it with a single command regardless of their preferred environment (Fig. 1B). For instance, an R user might call:

**Figure:**
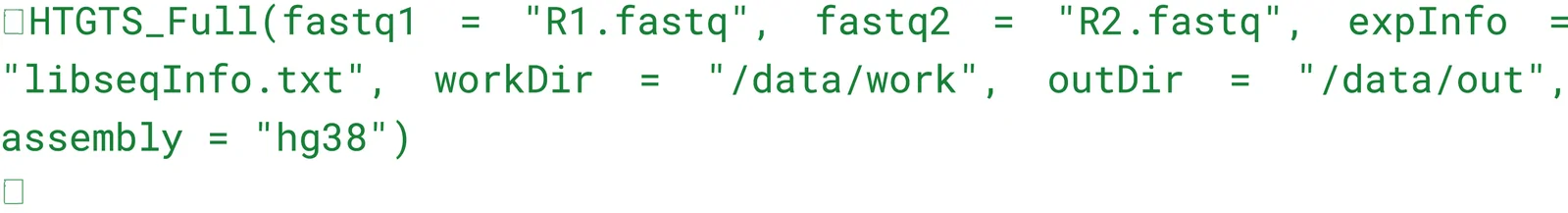

while a Python user would invoke the same analysis through:

**Figure:**
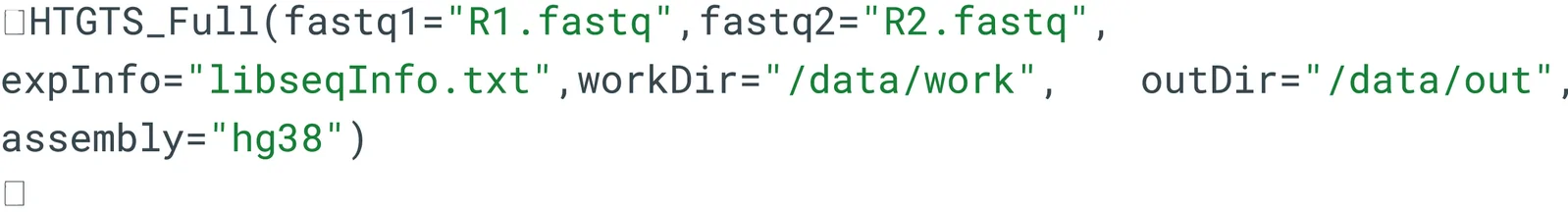

Both commands execute the identical containerized pipeline, ensuring reproducible results across languages and platforms. To maximize transparency and reusability, FairFlow-based pipelines should be distributed with their complete specification: the.bala file, generated wrappers, the container image or its DOI-archived TAR, and representative test data, ideally through version-controlled repositories and permanent archives such as GitHub and Zenodo.

### 2.5 Multi-step workflows are assembled from task-level units, each running in its own container

The HTGTS example above is a single computational unit: one Baryon file, one image, executed as a single command. Complex analyses are handled differently. Each task is described by its own Baryon file, so a workflow becomes a set of independent units rather than one wrapper around one image, and because each Baryon file declares its own container, different tasks run in different, independently pinned environments; tools with conflicting dependencies never have to coexist in the same image. For each task, FairFlow generates a unit that binds its own image: a Nextflow process carrying its own container directive, a Galaxy tool carrying its own container requirement, or a StreamFlow step bound to its own Docker deployment. Composition across tasks, including data passing, branching, scheduling and parallel execution, is performed by the workflow manager through its native constructs, not by FairFlow. A 15-step branched pipeline is therefore expressed as up to 15 Baryon files and up to 15 independently pinned images, with no monolithic container at any point. This task-level decomposition is used throughout CAR-NGS: the single-cell indexing (SCI) pipeline is split into two units that run in two different images, the bulk RNA-seq pipeline into five units (indexing and alignment, DESeq2, PCA, heatmap, and a downstream step), and the single-cell RNA-seq pipeline into units drawing on distinct alignment and downstream-analysis images (https://github.com/Fairflow-BioinformaticsFramework/CAR-NGS_Backend).

### 2.6 Galaxy deployment with lemaitre

For accessibility to non-programming users, FairFlow provides Lemaitre, an automated deployment service for Galaxy integration. The Galaxy tool XML is generated by the Baryon compiler from the.bala file; Lemaitre then installs it into the running Galaxy instance, handling container integration and providing drag-and-drop installation through a web interface. The galaxy-formed distribution (https://github.com/Fairflow-BioinformaticsFramework/galaxy-formed) provides a pre-configured, Baryon-compatible Galaxy server image with Lemaitre (https://github.com/Fairflow-BioinformaticsFramework/lemaitre) already integrated, so any FairFlow tool can be uploaded and made available without manual configuration.

### 2.7 HTGTS pipeline reconstruction and BLASTN version validation

All analyses were performed on previously published HTGTS sequencing datasets deposited in the Gene Expression Omnibus (GEO) database under accession GSE266237. The original study from which these data were derived obtained all necessary ethical approvals and informed consent as described in Tao et al., Blood, 2025 [13]. The original HTGTS analysis pipeline was reconstructed based on published code from Chiarle et al., 2011 [14] and Compagno et al., 2017 [15]. The pipeline performs the following steps: demultiplexing of raw FASTQ reads using primer/barcode sequences, alignment of demultiplexed reads to the reference genome, detection of translocation junctions based on split-read alignments, filtering of junctions by quality metrics. Within the HTGTS analysis pipeline, BLASTN is invoked during the demultiplexing and read-assignment steps, where sequencing reads are aligned against primer- or barcode-derived reference sequences. This step ensures that reads are correctly assigned to the appropriate experimental condition before downstream junction calling. The BLASTN version determines the set of alignments that pass through this demultiplexing stage, because the algorithm uses E-value thresholds and finite-size corrections that were modified in later releases. We verified this by re-running the pipeline with different BLASTN versions. Using BLAST+ 2.12.0 (2022), the demultiplexing step produced a different set of alignments compared to BLAST+ 2.2.31 (2015), despite identical parameters and input data. The divergence was traced to changes in the computation of E-values introduced after 2015, particularly in the finite-size correction module (see BLAST + release notes 2.4.0). For compatibility with the original HTGTS code, BLASTN 2.2.31 is required, and newer versions (≥2.4.0) cannot be substituted without altering the junction counts. For reproducibility, we encapsulated BLASTN 2.2.31 into a dedicated container image, based on the official repbioinfo/htgts_pipeline_lts_v16 but replacing only the BLASTN binary and its libraries. BLASTN was executed with the following parameters for demultiplexing:

**Figure:**
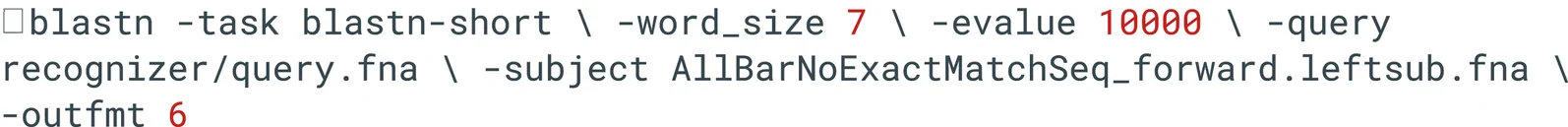

### 2.8 Parameter summary table generation

To document the effect of version drift in BLASTN, we generated a parameter summary table (Supplementary Table S1). For each query tested in the HTGTS demultiplexing step, the table records the top-scoring alignment statistics obtained with BLAST+ 2.2.31 (2015) and BLAST+ 2.12.0 (2022). Columns are as follows:

query: identifier of the primer, barcode, or bait sequence used for demultiplexing

label: orientation of the alignment (forward or reverse subject)

version: BLAST + release tested (2015 or 2022)

top_bitscore: bit-score of the highest scoring pair (HSP)

top_evalue: E-value reported for the top HSP

total_hits: number of alignments passing filters for the query

The table demonstrates that while bit-scores remain identical across versions, E-values differ substantially between 2015 and 2022 releases, consistent with the correction introduced in BLAST+ 2.4.0. In practice, this difference propagates into demultiplexing, causing discrepancies in the set of reads assigned to each barcode or primer and ultimately altering downstream junction counts.

### 2.9 Cross-platform reproducibility testing

To validate cross-platform reproducibility, the FairFlow-containerized HTGTS pipeline was executed on three different systems: Windows 11 (x86_64), macOS (ARM64, M1 chip), and Linux Ubuntu 22.04 (x86_64). System specifications are provided in Supplementary Table S2. On each system, the identical container image (referenced by SHA256 digest) was executed with identical input data and parameters. Junction detection results were compared evaluating percentage of shared junctions relative to total unique junctions.

### 2.10 CAR-NGS package development and validation

The CAR-NGS R package was developed entirely using the FairFlow framework. Each analysis pipeline (16S rRNA, ATAC-seq, bulk RNA-seq, Detect-seq, HTGTS, sci-RNA-seq, single-cell RNA-seq, whole-genome sequencing) was specified through a Baryon.bala file, and corresponding R wrapper functions were automatically generated using the Baryon compiler. The package was tested on Windows 11, macOS (M1), and Linux systems to verify cross-platform compatibility and reproducibility. Installation requires only R (any version) and Docker. The package is distributed through GitHub with comprehensive documentation and example datasets.

### 2.11 Quantification and statistical analysis

Concordance between HTGTS analyses was quantified as the percentage of shared translocation junctions relative to the total number of unique junctions detected across compared analyses. Junctions were considered identical if they matched in both genomic coordinates (chromosome, breakpoint position) and orientation.

**Fig. 2:**
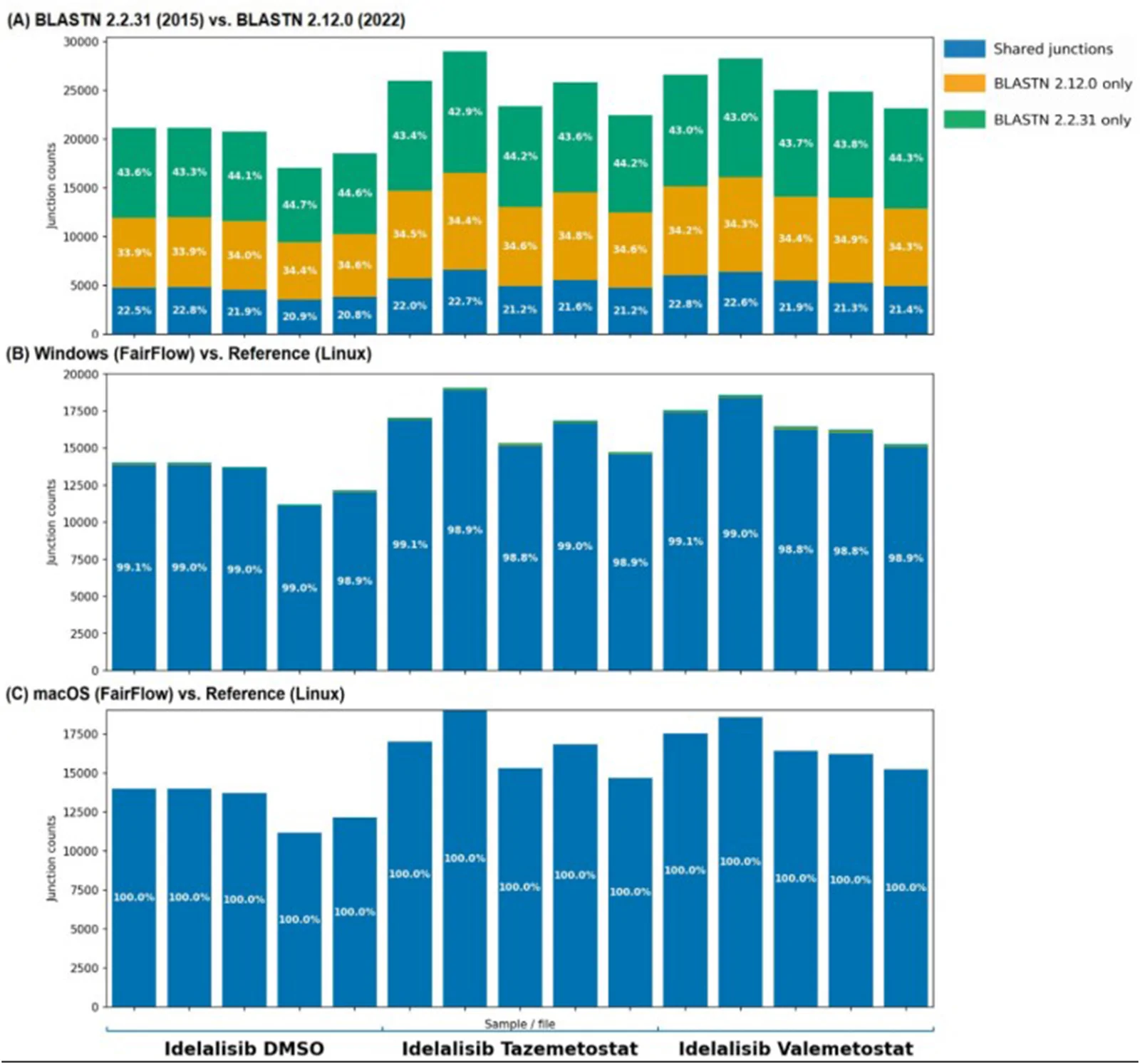
**HTGTS reproducibility depends on BLASTN version and is ensured by FairFlow.** (A) Comparison of HTGTS analysis using BLASTN 2.2.31 (2015) vs. 2.12.0 (2022), showing shared (blue), 2022-only (orange), and 2015-only (green) junctions. Concordance was calculated as the percentage of shared junctions relative to total unique junctions. (B–C) Reanalysis within FairFlow containers on Windows 11 and macOS using pinned BLASTN 2.2.31 environment.

For BLASTN version comparison (Fig. 2A), concordance was calculated as:

Concordance = (Number of shared junctions) / (Total unique junctions across both versions) × 100

For FairFlow cross-platform validation (Fig. 2B and C), concordance was calculated relative to the original 2015 analysis results:

Concordance = (Number of shared junctions with 2015 analysis)/(Total junctions in 2015 analysis) × 100

No statistical hypothesis testing was performed, as the study focused on exact reproducibility rather than statistical inference. All analyses were deterministic and executed with fixed random seeds where applicable. Sample sizes (n) refer to the number of independent system configurations tested (n = 3: Windows, macOS, Linux).

Statistical details for each analysis are reported in the corresponding figure legends.

## 3 Results

### 3.1 Unspecified BLASTN version caused 79% divergence in HTGTS translocation junction detection

The importance of standardization becomes even clearer in a real-case scenario. In a recent study [13], we analyzed HTGTS data (High-Throughput Genome-wide Translocation Sequencing, a method used to map chromosomal translocations) using the same code as in previous publications [14,15] but in a different computational environment. Reconstructing the environment proved extremely difficult: only a subset of the main tools had been declared as dependencies, while others were embedded in Java executables. After extensive troubleshooting, the pipeline ran without errors, yet the results significantly diverged from those reported in earlier papers with only ∼21% concordance in detected junctions (Fig. 2A).

The discrepancy was eventually traced to a single hidden dependency: BLASTN, a sequence alignment tool used during the demultiplexing step. The original publications had not specified which BLASTN version was used, so our analysis automatically installed the latest available release (2.12.0, from 2022) instead of the version that was current at the time of the original studies (2.2.31, from 2015). Although both versions accept identical parameters and run without errors, changes in the algorithm's internal E-value calculations, introduced in intermediate software releases, caused different sets of reads to pass the alignment filters, ultimately altering the detected junctions (detailed version comparison in Supplementary Table S1).

### 3.2 FairFlow containerization restored 98.8-100% concordance across operating systems

To ensure such errors cannot recur, we rebuilt the entire pipeline with FairFlow, encapsulating all dependencies, including the correct BLASTN version, into an immutable container. Reanalysis of the same HTGTS dataset with the FairFlow-based pipeline achieved 98.8–99.1% concordance on Windows and 100% on macOS (Fig. 2B and C), compared to the original ∼21% (Fig. 2A), with results remaining consistent across different operating systems and hardware architectures. Beyond numerical concordance, FairFlow dramatically reduced the barrier to reproducibility: reconstructing the original computational environment had required approximately three months of troubleshooting and dependency resolution, whereas executing the FairFlow-based pipeline on a fresh system required only Docker installation. The entire workflow, including data, code, and container specifications, is publicly available on Zenodo (https://doi.org/10.5281/zenodo.18599460), enabling independent verification and reuse.

### 3.3 CAR-NGS package validates FairFlow approach across eight production NGS pipelines with deterministic cross-platform execution

FairFlow was also extensively tested through CAR-NGS (https://github.com/Fairflow-BioinformaticsFramework/CAR-NGS), a comprehensive suite of pipelines for next-generation sequencing analyses, including 16S rRNA, ATAC-seq, bulk RNA-seq, Detect-seq, HTGTS, sci-RNA-seq, single-cell RNA-seq, and whole-genome sequencing, entirely built with our framework. Each R function within the package was automatically generated from its corresponding Baryon specification, ensuring consistent structure and reproducible behavior across systems. The package can be installed and executed identically on Linux, macOS, and Windows, requiring only Docker and R, as the generated R functions are self-contained and do not require Baryon at runtime. This was demonstrated by the HTGTS analyses presented in Fig. 2, where running the same command on each system yielded nearly identical junction-count results (≥99.8% overlap), with each machine executing the same container image with fixed seeds and deterministic defaults (detailed system specifications in Supplementary Table S2).

## 4 Discussion

The bioinformatics community has long lacked a shared standard for defining and distributing reproducible pipelines, resulting in a fragmented ecosystem where similar analyses are reimplemented independently across different systems. Imposing a single execution platform is neither realistic nor desirable, as different research contexts have genuinely different needs: individual researchers running single analyses may prefer simple R or Python functions, groups managing complex multi-step workflows rely on Nextflow or Snakemake, and non-programming users depend on graphical interfaces such as Galaxy. While existing tools address these needs well within their own domain, none provides a common specification layer that works across all of them. FairFlow was designed to fill exactly this gap, not as a replacement for these tools but as a complement that supports all of them simultaneously: from a single Baryon specification, it automatically generates the corresponding REIs, that is, programmatic functions for R and Python, a command-line script for Bash, an XML tool for Galaxy, or orchestration-ready configurations for Nextflow or StreamFlow. Table 2 summarizes how FairFlow and these systems compare across the criteria most relevant to reproducibility and accessibility. It is important to stress that this comparison is not meant to position FairFlow as a competitor of these tools: FairFlow does not replace Snakemake, Nextflow, or Galaxy, but operates as a complementary layer that, from a single specification, generates the REIs these systems then execute. The table therefore contrasts the properties each approach guarantees on its own, and shows where a common specification layer adds value rather than ranking alternative products.

**Table 2:** Reproducibility and accessibility criteria across bioinformatics workflow systems.

Criterion	FairFlow	Snakemake	Nextflow	Galaxy tools
Deterministic, time-stable execution environment	Yes	Partial^a^	Partial^a^	Partial^a^
Single specification for multiple platforms	Yes	No	No	No
Automatic generation of user-facing interfaces	Yes	No	No	No
Accessibility for non-programming users	Partial^b^	Partial^c^	Partial^c^	Yes
Reproducible execution across operating systems	Yes	Partial	Partial	Partial
Dependency from the tool itself	No	Yes	Yes	Yes

Comparison of bioinformatics workflow systems with respect to key criteria affecting computational reproducibility and accessibility. The table compares the properties each approach guarantees on its own, across environment stability, automatic interface generation, accessibility, and cross-platform reproducibility.

a Time-stable execution is achievable only if the pre-built container image is manually saved and archived; deterministic reconstruction of an identical environment from its recipe is not guaranteed.

b FairFlow enables accessibility for non-programming users through automatically generated graphical interfaces (Galaxy tools via Lemaitre).

c Snakemake and Nextflow are accessible to non-programming users only when a pre-configured pipeline is provided and run through a single command-line invocation, without a graphical interface.

Workflow managers such as Snakemake and Nextflow excel at orchestration and parallel execution and support containerization, but their reproducibility over time relies entirely on how developers manage their Docker environments. Pipelines are typically distributed with a Dockerfile rather than a pre-built image, and since Dockerfiles rely on package managers such as apt, pip, or conda that resolve dependencies dynamically at build time, the same Dockerfile built at different points in time may produce subtly different environments, making it impossible to guarantee identical results even with explicit version pinning as transitive dependencies remain unresolved and can change silently. Galaxy tools, conversely, offer excellent accessibility for end users through a graphical point-and-click interface, but adding new tools requires manually writing XML wrapper files tightly coupled to Galaxy's internal architecture, demanding considerable technical expertise and making it effectively inaccessible for the researchers who need to deploy or extend it.

FairFlow addresses these limitations by introducing a common specification standard through Baryon, where a single file describing what a pipeline needs, how it runs, and what it produces is sufficient to automatically generate the corresponding REIs for R, Python, Bash, Galaxy, Nextflow, and StreamFlow. To ensure long-term reproducibility, FairFlow guidelines recommend building execution environments with CREDO, a tool that, unlike standard Dockerfiles, freezes all transitive dependencies in a two-stage build process, ensuring that the same environment can be reconstructed identically across systems and over time. CREDO complements rather than replaces the distribution of pre-built images: because it produces both a ready-to-use image and a frozen, auditable build context, a FairFlow environment can be shared either as a CREDO-rebuildable specification or as a DOI-archived image TAR, the latter remaining a valid route when a pre-built image is the most practical option. A key practical advantage is that the REIs generated by FairFlow are self-contained and do not require FairFlow itself at runtime, meaning end users need only Docker and their preferred language, in contrast to Nextflow and Snakemake where users must install and maintain the workflow manager itself as a runtime dependency, introducing an additional software layer that can become a source of version drift and installation complexity.

The practical cost of lacking such a standard is clearly illustrated by the HTGTS case study (Fig. 2), where attempting to reproduce a published analysis with standard tools yielded only ∼21% concordance due to a single unspecified dependency version, and identifying the source of the discrepancy required approximately three months of troubleshooting (a scenario that is likely not isolated, as similar failures probably affect many published analyses where discrepancies go undetected simply because reconstructing the original environment is too difficult to attempt). After rebuilding the pipeline with FairFlow, concordance reached 98.8-100% across different operating systems and setup on a fresh system required only Docker installation, with the CAR-NGS package further validating this approach in a production setting across eight distinct NGS pipelines.

### 4.1 Limitations

While FairFlow substantially improves reproducibility and accessibility, several limitations should be acknowledged. The framework requires Docker or Singularity to be installed on the user's system, which may pose challenges in shared computing environments with restricted permissions or institutional policies prohibiting containerization, and users without administrative privileges may need to request IT support for installation. Pipeline developers also face a learning curve when transitioning from traditional scripting to declarative Baryon specifications, though this investment is substantially lower than mastering workflow managers like Nextflow or Snakemake, and we have attempted to mitigate it through comprehensive documentation and tutorials. FairFlow currently generates wrappers for a defined set of targets (R, Python, Bash, Galaxy, Nextflow, StreamFlow), and while the modular compiler architecture is designed for community extensibility, laboratories using other workflow systems such as Cromwell or Toil would need to contribute new compiler backends or adapt existing outputs. Finally, CREDO-based deterministic environments, while substantially more stable than standard Dockerfiles, may encounter edge cases with complex dependency graphs or proprietary software that cannot be freely redistributed, potentially requiring manual dependency specification and reintroducing some version drift risk. FairFlow should therefore be viewed as one component of a broader reproducibility strategy that includes detailed experimental documentation, raw data sharing, and transparent reporting.

## 5 Conclusions

Future development will focus on expanding compiler targets to additional workflow managers and languages and fostering community contributions to the Baryon ecosystem. A key planned improvement is the direct integration of CREDO into FairFlow, which currently provides it only as part of its development guidelines: by automating deterministic environment generation within the framework itself, the entire pipeline from specification to reproducible deployment would require no additional tooling, further lowering the barrier for developers building new pipelines. The broader goal is to make reproducible bioinformatics workflows the default rather than the exception, where published analyses are independently verifiable and reusable across laboratories without reimplementation.

## CRediT authorship contribution statement

**Agata D'Onofrio:** Software. **Eliseo Martelli:** Software. **Maurizio Alessandri:** Methodology, Software. **Sebastian Bucatariu:** Resources. **Sandro Gepiro Contaldo:** Validation. **Maria Luisa Ratto:** Formal analysis. **Jianli Tao:** Supervision. **Beatrice Nuvolari:** Resources. **Isabella Castellano:** Resources. **Andrea Loiacono:** Resources. **Sara Bianchi:** Resources. **Maddalena Arigoni:** Supervision, Validation. **Alessandro Bertero:** Supervision, Validation, Writing – original draft. **Elisa Balmas:** Supervision, Validation, Writing – original draft. **Roberto Chiarle:** Supervision. **Luca Alessandri:** Conceptualization, Data curation, Formal analysis, Investigation, Methodology, Project administration, Resources, Software, Supervision, Validation, Writing – original draft.

## Declaration of generative AI and AI-assisted technologies in the manuscript preparation process

During the preparation of this work, the authors used Claude (Anthropic) and ChatGPT (OpenAI) to improve the readability and language clarity of specific manuscript sections. After using these services, the authors reviewed and edited the content as needed and take full responsibility for the content of the publication.

## Funding

This work was supported by the Research program CN00000013 “National Centre for 10.13039/100019941HPC, Big Data and Quantum Computing”; GARR (Italian Academic and Research 10.13039/100022394Network) through a research fellowship to E.M.; and the 10.13039/501100000781European Research Council (10.13039/100017325ERC) under the European Union's 10.13039/100018693Horizon Europe research and innovation programme [grant agreement No. 101076026 to A.B.].

## Declaration of competing interests

The authors declare the following financial interests/personal relationships which may be considered as potential competing interests: Luca Alessandri reports financial support was provided by National Centre for HPC, Big Data and Quantum Computing. Eliseo Martelli reports financial support was provided by GARR (Italian Academic and Research Network). Alessandro Bertero reports financial support was provided by European Research Council (ERC). If there are other authors, they declare that they have no known competing financial interests or personal relationships that could have appeared to influence the work reported in this paper.

## Data Availability

The HTGTS datasets analyzed during the current study are available in the GEO repository, https://www.ncbi.nlm.nih.gov/geo/query/acc.cgi?acc=GSE266237 [13]. The complete FairFlow-based HTGTS workflow, including Baryon specifications (.bala files), Docker container images, and representative test data, is archived on Zenodo with DOI: https://doi.org/10.5281/zenodo.18599460. All FairFlow source code and CAR-NGS package are available at https://github.com/Fairflow-BioinformaticsFramework under MIT License.

## Appendix A Supplementary data

Supplementary Table S1 BLASTN version comparison showing algorithm changes affecting E-value calculations, related to Fig. 2A. Detailed comparison of BLASTN versions 2.2.31 (2015) and 2.12.0 (2022) highlighting specific algorithm modifications that affected junction detection in HTGTS analysis.

Supplementary Table S2 System specifications for cross-platform reproducibility testing, related to Fig. 2. Hardware and software configurations for Windows 11, macOS, and Linux systems used to validate FairFlow cross-platform reproducibility (≥99.8% junction concordance).

Supplementary Table S3 Software, data, and system resources used in this study. Names, sources, and identifiers of all software tools, container images, deposited datasets, and test systems.
